# Neoantigen-targeted TCR-engineered T cell immunotherapy: current advances and challenges

**DOI:** 10.1186/s40364-023-00534-0

**Published:** 2023-12-01

**Authors:** Zhi Pang, Man-man Lu, Yu Zhang, Yuan Gao, Jin-jin Bai, Jian-ying Gu, Lu Xie, Wei-zhong Wu

**Affiliations:** 1 Liver Cancer Institute, Key Laboratory of Carcinogenesis and Cancer Invasion, Ministry of Education, Zhongshan Hospital, Fudan University, Shanghai, 200032 China; 2 Clinical Center for Biotherapy, Zhongshan Hospital, Fudan University, Shanghai, 200032 China; 3 Shanghai-MOST Key Laboratory of Health and Disease Genomics, Shanghai Institute for Biomedical and Pharmaceutical Technologies, Shanghai, 200237 China

**Keywords:** Neoantigen, TCR-T, Neoantigen-reactive TCRs

## Abstract

Adoptive cell therapy using T cell receptor-engineered T cells (TCR-T) is a promising approach for cancer therapy with an expectation of no significant side effects. In the human body, mature T cells are armed with an incredible diversity of T cell receptors (TCRs) that theoretically react to the variety of random mutations generated by tumor cells. The outcomes, however, of current clinical trials using TCR-T cell therapies are not very successful especially involving solid tumors. The therapy still faces numerous challenges in the efficient screening of tumor-specific antigens and their cognate TCRs. In this review, we first introduce TCR structure-based antigen recognition and signaling, then describe recent advances in neoantigens and their specific TCR screening technologies, and finally summarize ongoing clinical trials of TCR-T therapies against neoantigens. More importantly, we also present the current challenges of TCR-T cell-based immunotherapies, e.g., the safety of viral vectors, the mismatch of T cell receptor, the impediment of suppressive tumor microenvironment. Finally, we highlight new insights and directions for personalized TCR-T therapy.

## Introduction

The efficacy of adoptive cell therapies (ACTs) with engineered TCRs depends mainly on identifying and using appropriate tumor antigens which specifically recognize T cells. Different tumor antigens, e.g. tumor-associated antigens (TAAs), tumor-associated viral antigens and tumor-specific antigens (TSAs), have been found [1, 2]. The former two, usually recruited in TCR-T immunotherapy, show some clinical efficacy in tumor-bearing patients. However, the on-target-off-tumor effects have largely limited their use in clinic.

During tumorigenesis and progression, numerous genetic abnormalities including point mutations, reading frameshift mutations, stop codon mutations, DNA insertions and deletions, or chromosomal translocations accumulate in tumor cells and produce many mutated peptides and proteins. Some of these mutants can activate T or B lymphocytes if they are hydrolyzed into shorter peptides and successfully presented by major histocompatibility complex (MHC). These immunogenic peptides are called neoantigens. Because neoantigens are not expressed in normal tissues, their specific T cells can escape negative selection in thymus and are therefore abundant in tumor patients with therapeutic potential [3].

Along with the rapid development of high-speed sequencing technologies in recent years, more and more TCR-T targeting neoantigens have been developed, but there are still many challenges. Therefore, in this review, we summarize TCR structure, activation and revisions, and introduce recent methodological advances in neoantigens and their cognate TCRs screening, and then summarize the ongoing clinical trials, their challenges, and the possible solutions for neoantigens based TCR-T immunotherapy.

## TCR structure-based T cell activation 

It is necessary to understand TCR structure and T cell activation at the cellular and molecular levels to fully understand how to initiate the most effective anti-tumor response, and why the immune response fails to eliminate tumor cells, as well as how to potentially modify the structure of TCR so that TCR-T cells can better kill tumors.

### TCR structure

**Fig. 1:**
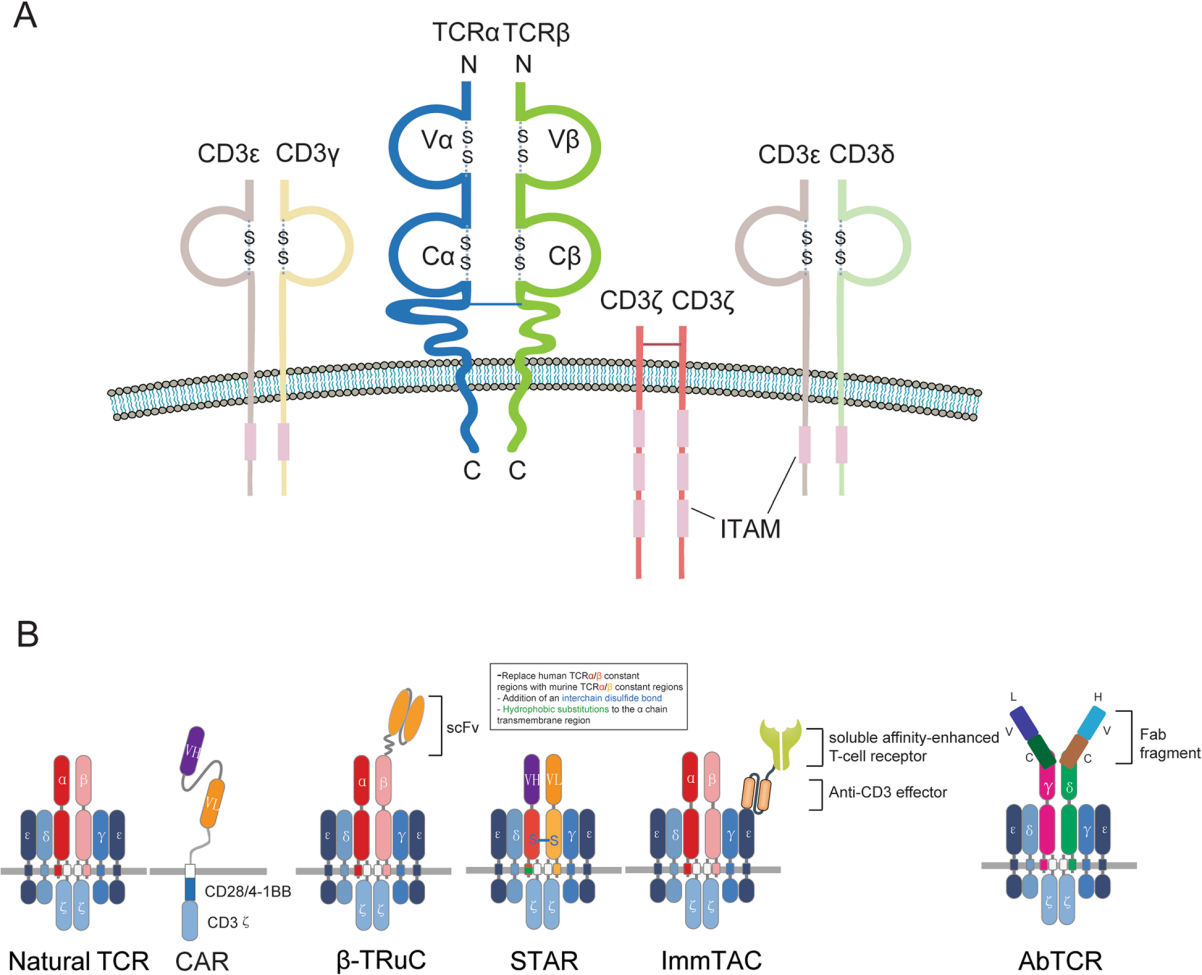
TCR structure **A** TCR-CD3 complex and ITAM. Cysteine (S) mediates interchain bridge of disulphide. **B** The natural TCR complex, CAR and four recombinant TCR complexes

**Fig. 2:**
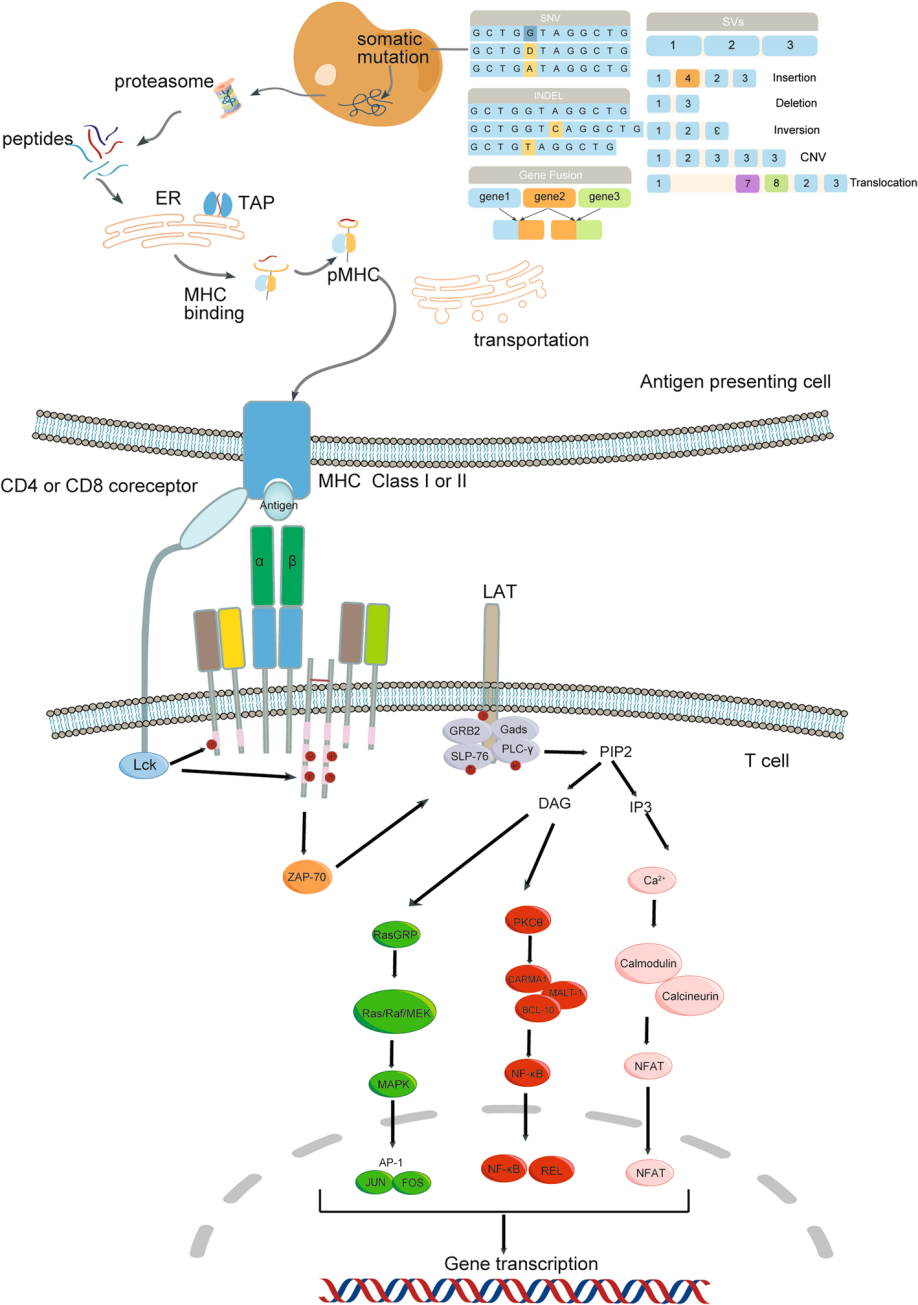
Neoantigen presentation and regulatory mechanisms in T cell receptor signaling Neoantigen is generated by tumor cell genome mutation, transcribed and translated and cleaved to peptides different from normal self-proteins. Immunogenic neoantigen peptides are bound by MHC molecules (pMHC), and required for recognition by TCR and to initiate immune response. TCR signal is initiated by pMHC recognition of tumor cells or antigen-presenting cells. Then Lck is recruited to TCR-CD3 complex and phosphorylate ITAMs. Zap70 binds to phosphorylated ITAMs and is also phosphorylated itself by Lck. Activated ZAP70 subsequently phosphorylates Lat, which in turn induces the recruitment of adaptor proteins (GRB2, Gads, SLP-76, PLC-γ). Activation of LAT-related effectors results in signal transduction through 3 major signaling pathways. Calmodulin, MAPK and NF-кB signaling pathways. Calmodulin signaling leads to nuclear translocation of NFAT. MAPK signaling leads to actin polymerization and AP-1 activation, a transcription factor of FOS/ JUN complex. NF-кB signaling leads to nuclear translocation of transcription factors of REL and NF-кB

In human being, there are two types of TCR’s, namely αβ TCR and γδTCR. The former predominates. TCR forms an octamer comprised of an antigen-binding subunit (TCRαβ) with three CD3 signaling subunits (CD3δε, CD3γε and CD3ζζ). CD3γ, CD3δ and CD3ε chains, each containing an immunoreceptor tyrosine-based activation motif (ITAM), while CD3ζ chain contains 3 ITAMs. The entire TCR-CD3 complex contains a total of 10 ITAMs [4] (Fig. 1A). Tyrosine phosphorylation in these ITAMs plays an important role in TCR signaling. Phosphorylation of ITAMs by the Src family kinase Lck initiates downstream T cell signaling (Fig. 2). Thus, the TCR-CD3 complex remains structurally intact while developing new TCR chimeric structures. By activating the extracellular region of the TCR complex, it can activate T cells and transmit the signal downstream. Liang et al. recently reported a novel mechanism by which cholesterol sulfate (CS) interacted with the cytoplasmic domain of CD3ε to enhance its binding to the cell membrane and induce a stable secondary structure. This structure inhibited TCR phosphorylation and signaling. When a point mutation (I/A) was introduced to the ITAMs of CD3ε, it would reduce the stability of the secondary structure, abolish CS-mediated inhibition and enhance the signaling of the TCR complex [5]. For the first time, this study realized the rational design of signal-enhanced TCR-T cells by revealing the signal regulation mechanism of TCR/CD3 complex signaling, which laid a solid theoretical foundation for further improving the efficacy of immune cells in solid tumors in the future. As to the structure of γδTCR, readers can consult the excellent review of Legut M [6].

### T cell activation

If T cells encounter and bind to peptide major histocompatibility complexes (pMHCs) in antigen-presenting cells (APCs), a TCR activation program is initiated [7]. TCR recognizes pMHC in a manner of "immunological kinapse" (IK) or "immunological synapse" (IS). Kinapse is a transient and unstable structure while synapse is stable long-term [8]. T cell activation and signaling depends on a continuous contact of the TCR with pMHCs. Sufficient activation of T cells requires three signals. One is antigen-specific signal via TCR-pMHC complex; the other is a costimulatory signal like CD28-B7 (CD80, CD86). Cytokines act as a third messenger, which provide cell proliferation and survival signals in activated T cell [9]. Therefore, enhancing costimulatory signals or increasing cytokine action is another way to improve TCR-T cell function (discussed in more detail in Sect. Challenges of neoantigen-based TCR-T therapies).

### Recombinant TCRs

As we know, Chimeric antigen receptor (CAR)-T cell therapy utilizes a synthetic receptor capable of recognizing specific antigens on the surface of cancer cells, such as CD19. Due to its inherent high specificity, CAR-T cell therapy has been successful in the treatment of hematologic malignancies like acute lymphoblastic leukemia but has shown limited efficacy in solid tumors. Unlike CAR-T's single receptor, TCR-T immunotherapy employs the natural T-cell receptor (TCR) to identify specific tumor antigens. And in this recognition process, T-cell activation is more selective and regulated, thereby reducing the risk of excessive activation and cytokine release. However, in practice, antigen loss and down-regulation of MHC molecules often occur in tumors. In addition, allogeneic application of TCR-T therapy is limited due to the individuality of MHC types. In order to overcome these limitations, rapid advances of TCR structural modifications to improve immunotherapy efficiency have been made, such as STAR, AbTCR, ImmTAC et al. (Fig. 1B).

Synthetic T cell receptor and antigen receptor-T (STAR-T) integrates the advantages of CAR-T and TCR-T, is an MHC-independent high-affinity TCR-T. STAR is an antibody-TCR chimera, in which TCR constant regions are ligated with variable regions of heavy and light chains of antibody. In order to maintain natural TCR signaling, gene mutation and addition of functional elements can be performed on the constant and intracellular regions of TCR. For example, human TCR constant region-based STAR (hSTAR) can be optimized as mutSTAR. The mutSTAR has high affinity, specificity and is MHC-unrestricted to surface antigen [10]. T cell receptor fusion constructs (TRuCs) are comprised of an antibody-based binding domain (single-chain variable fragment, scFv) fused to one of the TCR subunits, which can recognize tumor surface antigens effectively via reprogramming TCR complex and kill tumor cells independent of MHC [11]. TCR mimic (TCRm) antibodies have been shown to mimic the specificity of TCR for peptide/MHC class I complexes and mediate antibody-dependent cytotoxicity [12]. Liu et al. constructed a novel TCRm antibody that recognizes alpha-fetoprotein polypeptide/HLA-A*02 complex, which has the function of TCR and can target intracellular antigens of hepatoma cells. The Fab fragment is fused to the γ and δ subunits of the TCR to form an antibody-T cell receptor (AbTCR) structure capable of transmitting a signal. At the same time, a scFv/CD28 co-stimulatory molecule targeting phosphatidylinositol proteoglycan 3 (glypican-3, GPC-3) was constructed. AbTCR and co-stimulatory molecule were delivered to T cells by a lentiviral vector. The proliferation and activation of T cells were enhanced through AbTCR signaling and CD28 co-stimulated signaling [13, 14]. In addition, immune-mobilizing monoclonal T cell receptors against cancer (ImmTACs) are bifunctional reagents that combine a soluble TCR with affinity for an intracellular or extracellular tumor-specific antigen and an anti-CD3 scFv antibody. These ImmTACs redirect T cells specifically toward tumor cells presenting a target peptide-MHC complexes [15]. Boudousquie et al. reported the IMCgp100, an ImmTAC recognizing a peptide derived from the melanoma-specific protein, gp100, efficiently redirects and activates effector and memory cells from both CD8^+^ and CD4^+^ T cells. The IMCgp100 induces broad immune responses [16].

## Neoantigen and cognate TCR prediction and screening strategies

Although a series of clinical trials of engineered TCR-T cells have been carried out, tumor antigens available for TCR-T therapy remain very limited (discussed in more detail in Sect. Neoantigen-targeted TCR-T therapy in clinical trials). Since neoantigens with therapeutic potential are critical for anti-cancer immunotherapy, the prediction and selection screening of tumor neoantigens are essential. Numerous neoantigens have been discovered through high-throughput sequencing and computational prediction, some have been tested in immunotherapy clinical trials of cancer patients. With the aid of protein level verification data, neoantigen and its cognate TCRs in silicon predictions have become more precise, even more so as in vitro or in vivo experimental validation are optimal for clinical trials.

### Computational prediction of neoantigen peptide binding to MHC

The fundamental premise for an immune response to occur is that the mutated peptide is effectively bound to and presented by MHC molecules to elicit a robust immune response. Therefore, predicting the probability of MHC molecules binding to peptides is a key step in current computational pipelines. Published MHC binding affinity prediction algorithm integrates ligand datasets into a machine learning algorithm and utilizes the receiver operating characteristic (ROC) curve to evaluate the likelihood of peptide binding or presentation, including NetMHC 4.0 [17], MHCflurry [18], MixMHCpred [19], etc. NetMHCpan is an advanced MHC binding affinity prediction algorithm that is trained using affinity measurements and mass spectrometry (MS) elution data of MHC ligands. By leveraging homology with well-characterized MHC alleles, the algorithm infers potential ligand preferences, ensuring its robustness and effectiveness when compared to other prediction tools [20].

New studies have highlighted the crucial importance of collaborative interactions between antigen-specific CD4^+^ and CD8^+^ T cells in anti-tumor immunity. Consequently, for effective anti-tumor immune response, consideration should be given to neoepitopes that can bind to the MHC-II alleles of the individual patient. Artificial neural networks have been widely used in the development of prediction tools for MHC-II binding epitopes, including NetMHCII [21], NetMHCIIPan [20], SMMAlign [22] and NNalign [23] are used for predicting MHC-II binding peptides (Table 1). Indeed, prediction of neoantigens presented by MHC-II remains challenging compared to the accuracy of Class I tools. Firstly, the peptide-binding groove of MHC-II is relatively shallow and open on both sides, leading to a wide variation in the length of binding peptides (9 to 22 residues) [24]. Secondly, the polymorphism of the α and β chains in MHC-II molecules has further expanded the diversity of peptide binding specificity [25]. Thirdly, the availability of data on validated binding to MHC-II class molecules is limited, making it challenging to train and validate prediction models accurately. In light of the above, given that no predictive tool consistently performs well across all peptide lengths and all HLA classes, some predictive tools can simultaneously combine different algorithms to predict the binding presentation of MHC molecules, thereby improving overall performance.

**Table 1:** Current computational pipelines for neoantigens prediction and screening

Prediction tool Pipeline	Time	Input data type	Workflow and Features	HLA allele	MHC-peptide	Validation in vivo or in vitro
INTEGRATE-neo	2016	SV	Starting from the original FASTA/FASTAQ file, the main steps included prediction of fusion gene peptides, prediction of HLA alleles, and prediction of neoantigens	HLAminer	NetMHC4.0	No
[26]						
TSNAD/ TSNAD v2.0	2017/2021	SNV, indel / SNV, indel, fusion	TSNAD is a one-stop software solution for predicting neoantigens from WES/WGS data of tumor-normal pairs. The new version adds RNA-seq data analysis, supports two versions of the reference genome (GRCh37 and GRCh38), uses DeepHLApan instead of NetMHCpan, and provides Web services and Docker installation methods	OptiType	DeepHLApan	No
[27, 28]						
MuPeXI	2017	SNV, indel	MuPeXI acceptes WES/WGS and RNA-seq sequencing data as source data. MuPeXI measures the immunogenicity of new antigens based on quantitative scores of important features of new peptides, prioritizing predicted peptides based on affinity of mutant and normal peptides, allele frequencies of mutant peptides, and gene expression levels	OptiType	NetMHCpan4.0	Clinical verifications [30–32]
[29]						In vivo validation [33]
						In vitro validation [34]
CloudNeo	2017	SNV, indel	CloudNeo is a cloud-based workflow for identifying tumor neoantigens in patients, which is a common workflow language implementation of HLA typing using Polysolver or HLAminer combined with custom scripts for mutant peptide identification and NetMHCpan for neoantigen prediction	HLAminer/Polysolver	NetMHCpan3.0	No
[35]						
Timiner	2017	SNV, indel	TIminer integrates a suite of bioinformatics tools to analyze RNA-seq data and somatic DNA mutations from a single sample, including: 1) HLA genotyping from NGS data; 2) Using mutation data and HLA types to predict tumor neoantigens; 3) Identification of tumor infiltrating immune cells from RNA-seq data; 4) Analysis of tumor immunity profile from expression data	Optitype	NetMHCpan3.0	No
[36]						
pTuneos	2019	SNV, indel	pTuneos presents a computational framework for tumor de novo antigen sequencing and screening to effectively predict neoantigens from high-throughput sequencing data and evaluate and rank their true immunogenicity. Moreover, pTunes architecture implements multi-thread processing, which improves the speed of high-throughput sequencing data processing	OptiType	NetMHCpan4.0	No
[37]						
Neo-Fusion	2019	SV	Neo-Fusion utilizes two separate ion database searches to identify the two halves of each spliced peptide and infer the complete spliced sequence. The feature of this tool is that it allows the identification of spliced peptides without the restriction of peptide length, providing a widely applicable tool for the identification of spliced peptides	OptiType	NetMHCpan	No
[38]						
NeoPredPipe	2019	SNV, indel	Allow users to process neoantigens predicted from single or multi-region vcf files using ANNOVAR and NetMHCpan	POLYSOLVER	NetMHCpan	No
[39]						
NeoFuse	2020	Fusion	NeoFuse is a computational pipeline for the prediction of fusion neoantigens from tumor RNA-seq data, which is available as Singularity and Docker images to simplify installation and analysis	OptiType	MHCflurry	No
[40]						
NeoFlow	2020	SNV, indel	NeoFlow consists of four functional modules: (1) variation annotation and construction of sample personalized protein database; (2) Identification of peptides based on mass spectrometry data; (3) HLA type prediction based on WGS data or WES data; (4) Neoantigen prediction. This process can also be used to analyze data from immune polypeptide groups	-	-	No
[41]						
OpenVax	2020	SNV, indel	OpenVax is a computational workflow for identifying somatic mutations, predicting neoantigens, and developing personalized cancer vaccines. OpenVax is also an end-to-end workflow that starts with raw DNA and RNA FASTQ data, generates a mutant containing peptide, and finally outputs a specified length of synthetic growth peptide containing the mutant peptide segment	-	Different options	NCT02721043
[42]						NCT03223103
						NCT03359239
pVACtools	2020	SNV, indel,fusion	pVACtools is an integrated computing tool pipeline composed of five main parts, including pVACseq, pVACbind, pVACfuse, pVACvector, and pVACviz, which is highly modularized and is divided into flexible components that can be run independently. pVAC-tools can support the identification of mutant peptides from from different sources, including missense, frameshift mutations, insertions-deletions, and gene fusions. The predicted peptides were prioritized by integrating different data, including the expression of the mutant allele, the affinity of the binding peptide, and the type of clone. pVACtools has been used to predict and develop cancer vaccines for immunotherapy research in clinical trials	HLAminer/Athlates	8 MHC Class I algorithms: NetMHCpan, NetMHC, NetMHCcons, PickPocket, SMM, SMMPMBEC, MHCflurry, and MHCnuggets;	NCT00683670 [43]
[44]					4 MHC Class II algorithms: NetMHCIIpan, SMMalign, NNalign, and MHCnuggets	NCT03199040 [45] NCT02510950 [46]
						In vitro validation [47, 48]
						In vivo validation [49]
ASNEO	2020	SV	ASNEO is an integrated computational pipeline that analyzes RNA-seq data to identify neoantigens generated by personalized alternative splicing	OptiType	NetMHCpan4.0	No
[50]						
Neoepiscope	2020	SNV, indel	Neoepiscope is used to predict epitopes from DNA-seq data, which can incorporate germline context and address variant phasing for SNVs and indels. Neoepiscope framework is sufficiently flexible to accommodate numerous variant types, nonsense-mediated decay products and epitope prediction across different genomes	Optitype,PHLAT	MHCflurry, MHCnuggets or independently install NetMHCpan and NetMHCIIpan	No
[51]						
neoANT-HILL	2020	SNV, indel	neoANT-HILL integrates multiple immunogenomic analyses combined with quantification of immune cells infiltrating tumors and considers the use of RNA-Seq data to identify potential de novo antigens. In particular, neoANT-HILL is a user-friendly software tool with a graphical interface that can be used by users without programming skills	Optitype	Employs seven binding prediction algorithms: NetMHC, NetMHCpan 4.0, NetMHCcons, NetMHCstabpan, PickPocket, SMM and SMMPMBEC, MHCflurry	No
[52]						
TruNeo	2020	SNV, indel,	TruNeo pipeline requires to input raw DNA sequencing and RNA-seq data. The prediction steps include annotating somatic mutation information, obtaining HLA genotype and gene expression information; Candidate neoantigens were predicted according to the affinity of peptide binding to MHC. The candidate neoantigens were scored by integrating the information of multiple neoantigen presentation processes, and the high confidence neoantigens were finally screened and output	Polysolver and BWA-HLA	NetMHCpan3.0	In vitro validation [53]
[54]						
ProGeo-Neo v2.0/ProGeo-Neo	2022	SNV, indel, fusion	ProGeo-neo v2.0 is a proteogenomics-based neoantigen prediction pipeline, a one-stop software, which is divided into five modules: WES/WGS data processing; RNA-seq data processing; The construction of customized mutant protein sequence database and the identification of MS data database; Prediction of neoantigens; Computational screening of neoantigens	OptiType	NetMHCpanI 4.1	No
[55, 56]					NetMHCpanII 4.0	
PGNneo	2023	Noncoding regions	PGNneo is a proteogenomics-based pipeline developed for predicting neoantigens from non-coding regions. The pipeline consists of four modules: (1) non-coding somatic variant calling and HLA typing; (2) peptide extraction and customized database construction; (3) identification of variant peptides; (4) selection of candidate neoantigens	OptiType	NetMHCpan 4.1	No
[57]						

*Abbreviations SV* Structural variation, *SNV* A single-nucleotide variant indel, an insertion or deletion of bases in the genome, *NGS* Next-generation sequencing

### Computational prediction of TCR-pMHC binding

In recent years, one great advance in neoantigen prediction has shifted from focusing solely on the antigenic peptide to its interaction with T cell receptor (Table 2). De Neuter et al. first used random forest classifiers and discovered both the length of TCR sequence and the number of arginines within TCR complementarity determining region 3(CDR3) that affect T cell recognition [58]. Gielis et al. proposed a novel strategy to annotate full TCR repertoires with their epitope-binding specificities, which has been validated in three independent datasets. The antigen-specific TCR repertoires were increased post-vaccination [59]. The application of artificial intelligence in protein structure prediction can effectively utilize sequence and structural information to construct novel deep learning network architectures. Natural Language Processing (NLP) based methods can be applied to predict TCR-binding peptide from large-scale dictionaries. Springer et al. constructed models ERGO-AE and ERGO-LSTM, which were trained using autoencoder (AE) and long short-term memory (LSTM), respectively [60]. Moris et al. presented a novel interaction map recognition (imRex) method that can be used to predict previously unseen epitopes. ImRex demonstrated superior performance on known epitopes and showed the ability to infer epitopes that are more similar to the training data than standard dual input methods [61]. All models mentioned above can only support peptide and TCR β-chain sequences. However, it was reported that the α-chain of TCRs can also contribute to binding specificity. Xu et al. described a model, DLpTCR, which was constructed using ensemble deep learning for single/paired chain(s) of TCR and peptide interaction prediction [62]. Additionally, MHC proteins should also be included in epitope prediction as they were thought to affect the spatial locations of the epitope anchor positions [63].

**Table 2:** Currently available TCR-epitope binding prediction methods

TCR-epitope binding prediction tool	Predictable TCR chain(s)	Epitope constraint	TCR length constraint	Method description	Published date	Software / Webserver
**TCRex**	TCR β	Restricted epitopes	None	Models based on random forest classifiers	Nov-2019	Webserver:
						https://www.tcrex.biodatamining.be/
**ERGO-LSTM**	TCR β	None	None	LSTM based model	Aug-2020	Software:
						https://github.com/louzounlab/ERGO
**ERGO-AE**	TCR β	None	None	Autoencoder based model	Aug-2020	Software:
						https://github.com/louzounlab/ERGO
**ImRex**	TCR β	8 ~ 11-mer	10 ~ 20	Dual input CNN model	Dec-2020	Software:
						https://github.com/pmoris/ImRex
**DLpTCR**	TCR αβ	9-mer	8 ~ 20	Ensemble deep learning model of FCN, LeNet-5 and ResNet	Jul-2021	Software:
						https://github.com/jiangBiolab/DLpTCR
						Webserver:
						http://jianglab.org.cn/DLpTCR/
**NetTCR-2.0**	TCR αβ	9-mer	8 ~ 18	1-dimensional CNN model	Sep-2021	Software:
						https://github.com/mnielLab/NetTCR-2.0/
						Webserver:
						https://services.healthtech.dtu.dk/service.php?NetTCR-2.0
**pMTnet**	TCR β	None	None	deep neural network based on LSTM and stacked autoencoders	Sep-2021	Software:
						https://github.com/tianshilu/pMTnet

*Abbreviations LSTM* Long short-term memory, *CNN*,Convolutional neural network, *FCN* Fully convolutional networks, *ResNet* Residual neural network

To discover unknown structural drivers of T-cell activation and design novel peptide ligands and vaccines, it is important to understand the peptide binding details of the spatial conformation of TCR-pMHC [64–66]. Unfortunately, only a few 3D structures of pMHC complexes and TCR-pMHC are available in the Protein Data Bank [67–69]. Lack of information on the common binding site and orientation for a given peptide, as well as its correct docking in TCR-pMHC complexes are still significant challenges for predictive structural modelling approaches. Although Alphafold2, an artificial intelligence tool, appears to provide superior protein structure prediction [70], its prediction accuracy of TCR-pMHC binding conformation needs to be further validated.

### Comprehensive pipelines of neoantigens prediction in silicon

**Fig. 3:**
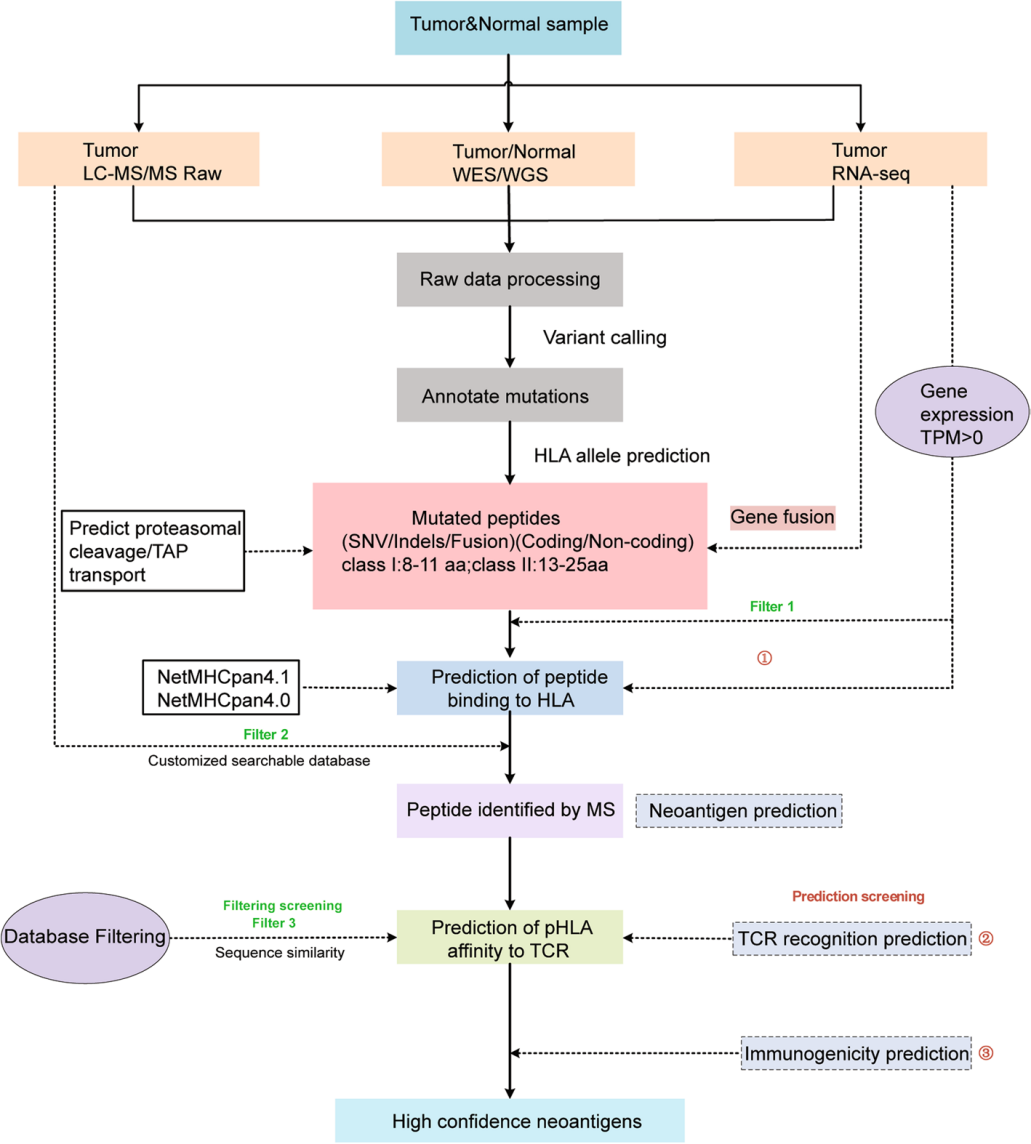
The workflow of computational prediction and screening pipelines for neoantigens To identify tumor-specific somatic mutations, tumor tissue and normal tissue samples (usually peripheral blood mononuclear cells) are acquired from the patient perform WES/WGS. Additional RNA sequencing provides information on the gene expression of the mutated genes and further confirmation of gene fusion. Peripheral blood cells were used to predict HLA typing performed by RNA-seq or DNA-seq analysis. MHC-peptide binding prediction software predicts peptides presented by MHC. Computational filtering/screening involves three levels: filter 1 is based on RNA expression; filter 2 is based on proteomics mass spectrometry identification; filter 3 is based on database high confidence filtering. By integrating various physical and chemical properties of peptides, computational prediction screening also includes three levels: antigen binding; neoantigen peptide epitope-TCR recognition; immunogenicity calculation to prioritize the predicted peptides and screen out the neoantigens with high confidence that could be recognized by TCR

Typically, the prediction of neoantigens begins with the identification of all somatic mutants from the whole exome/genome sequencing of tumor samples [31]. However, not all mutations lead to effective neoantigen products. To identify neoantigens capable of activating T cells, the prediction of neoantigens needs to consider factors such as mutation type, proteasome degradation, transporter associated with antigen processing (TAP), HLA molecule binding and presentation and the recognition potential of the T cell receptor (Fig. 2). The existing classical complete workflows for neoantigen prediction can be summarized in the following steps:1) perform whole-exome sequencing (WES) of peripheral blood monocytes or normal tissue and tumor tissue to identify tumor-specific mutated peptides; 2) Analyze HLA typing by RNA-seq or DNA-seq in peripheral blood cells; 3). Predict affinity between mutant peptides and MHC molecules; 4) Prioritize TCR recognition of the candidate peptides. Currently, numerous valuable bioinformatics tools have been established for each step, thus utilizing various combinations of algorithmic tools, the key parameters affecting the selection and prioritization of neoepitopes can be determined. Such optimal combinations may form effective comprehensive pipelines for neoantigen prediction in silicon [26–29, 35–42, 44, 50–52, 54–56] (Fig. 3), such as TSNAD, TIminer, MuPeXI, Neo-Fusion and pVACtools. Some of the predicted neoantigen epitopes have shown promising results in clinical trials [43, 45–48] (Table 1). For example, one glioblastoma patient was inoculated with synthetic eight amino acid peptide (SLP) vaccines produced by the pVAC-seq predictive pipeline (NCT02510950) [49], and three HLA I as well as five HLA II restricted neoantigens were detected in peripheral blood by IFN-γ enzyme-linked immunospot (ELISPOT) after vaccination. In vitro, 52 neoantigens inducing CD8^+^ T cell-specific responses were detected by MuPeXI in six patients with clear cell renal cell carcinoma (ccRCC) [30, 32–34, 71]. The OpenVax pipeline can produce SLPs with user-specified lengths and three SLP vaccines with long mutant peptides have been tested respectively in phase I clinical trials (NCT02721043, NCT03223103 and NCT03359239) [42, 53].

Integrated analysis of genomics and proteomics, proteo-genomics, in theory, may more accurately identify real genomic to proteomic alterations of somatic mutations in cancer cells [72]. Among these, MS-based approaches are considered appropriate for directly analyzing immunopeptides that are actually presented, providing protein level verification against HLA-binding neoantigens predicted solely on genomics data. The incorporation of mass spectrometry data has made the prediction algorithms and prediction pipelines more diverse, the output list of predicted neopeptides shorter but more reliable, such using NeoFlow, ProGeo-neo [41, 56] (Table 1). Advances in proteogenomic approaches not only extended neoantigen prediction pipelines for neoantigens derived from coding regions, but also help generated non-coding neoantigen prediction pipeline, such as PGNneo [57].

### Neoantigen selection screening strategies in silicon

Up till now the neoantigens are still predicted computationally from pipelines. Further selection approaches which we call screening are vital to identify neoantigens with greater potential to generate immune response in TCR-T related immunotherapy. Such screening may be computational or experimental. In silicon screening is the first step. Immunogenic features have been found to be associated with T cell activation, including sequence similarity, peptide entropy, peptide-binding residues, physicochemical properties of amino acids, molecular structure, and sequence length [73, 74]. Integrating these potential immunogenic features into the pipeline for neoantigen computation can enable a more precise assessment of the immunotherapeutic efficacy of the identified neoantigens. Computational screening strategies include data filtering and algorithm screening strategies (Fig. 3). Computational filtering/screening includes three levels: filter 1 is based on RNA expression; filter 2 is based on mass spectrometry identification; filter 3 is based on database high confidence blast search [75, 76]. Neoantigens with sequence similarity above a defined threshold are more likely to be immunogenic. Algorithm screening involves three levels: antigen binding; neoantigen peptide epitope-TCR recognition; immunogenicity calculation. Computational screening strategies are more or less imbedded in almost all above mentioned neoantigen prediction pipelines. Such screening may narrow down the candidate neoantigen list for further real experimental validation, and help improve the success of clinic trials.

### Experimental screening strategies for tumor neoantigens

**Fig. 4:**
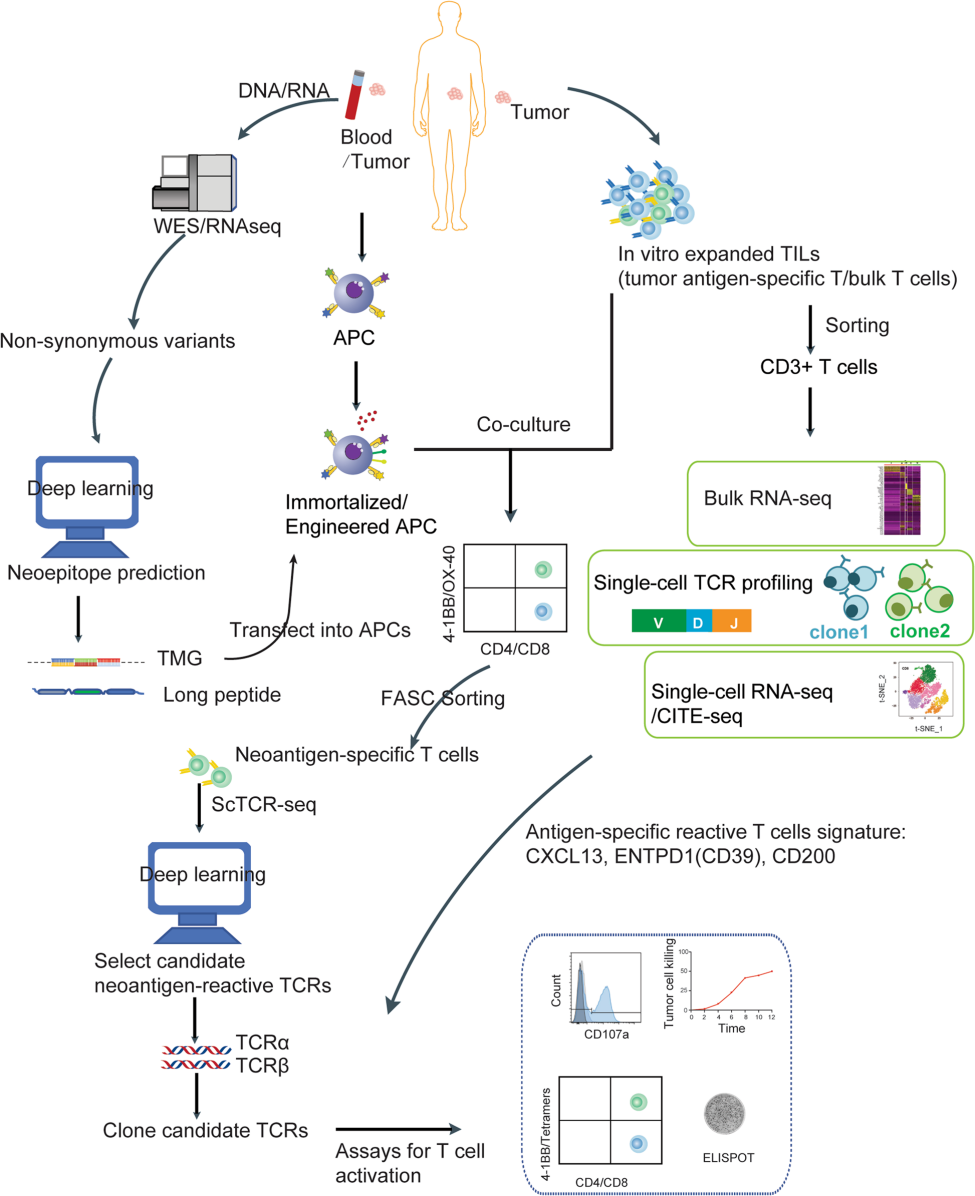
Schematic overview and validation of neoantigen and cognate TCR discovery technology. Tumor and/or peripheral blood mononuclear cell (PBMC) derived DNA/RNA are used to perform WES/RNA-seq to identify non-synonymous variants. Through deep learning-based prediction of neoantigen epitopes, select candidate epitopes to synthesize TMG/long peptides. The monocyte-derived APCs should be engineered to promote antigen presentation and T cell activation. Then, immortalized/engineered APCs were loaded with antigen library. When APCs co-cultured with tumor-infiltrating lymphocytes, neoantigen-reactive T cells will be labeled and selected by flow cytometry. The neoantigen-specific TCR are screened by scTCR-seq, and clone candidate TCRs to PBMC derived T cells. Finally, the recognition of neoantigens by T cells is verified by several screening experiments, such as neoepitope tetramers/4-1BB staining, IFN-γ ELISPOT, cytotoxic activity of tumor killing, degranulation. Meanwhile, neoantigen-specific TCRs could be rapid cloned through T cell characterizing by a panel of CXCL13, ENTPD1(CD19) and CD200 etc.

Accurately assessing the potential of neoantigens in immunotherapy and experimentally validating their reactivity with T cells remain the gold standard for clinical selection. T cells co-cultured with autologous APCs which loaded with different potential peptides is the most direct method to detect the reactogenicity of tumor antigens. At present, potential peptides are introduced into cells simultaneously in the form of tandem mini-gene (TMG) or a long peptide to improve the screening efficiency [77]. Although successfully used in clinical studies of various solid tumors, including colorectal tumor, melanoma and lung cancers [77, 78], these two methods are arduous and time-consuming for their requirement of multiple screening rounds to identify reactive tumor antigens [77]. A combined use of highly diverse yeast-displayed peptide-MHC libraries and deep sequencing largely expands the numbers of recognized epitopes [79, 80]. Alternatively, peptides can be genetically encoded and displayed on cell surface as pMHC complexes in baculoviral libraries. For example, pMHC complexes were anchored on Sf9 cell membrane by fusing them with a transmembrane domain of baculovirus gp64 molecule [81]. The engineering of APCs can further improve the efficiency of neoantigen screening. Arnaud et al. developed a method called Neoscreen, based on exposure of tumor-infiltrating lymphocytes (TILs) to CD40-activated (CD40-act) B cells that optimizing the sensitivity of antigen validation. CD40-act B cells expressed key molecules required for antigen presentation and T cell activation, such as IL-2, OX40L and 4-1BBL. And CD40-act B cells loaded with diverse sources of neoantigens (that is, transfected with minigenes or pulsed with synthetic peptides) ensured efficient stimulation of neoepitope-specific CD8 TILs ex vivo [77]. Recently, Cattaneo et al. proposed a high-throughput genetic system for personalized identification of CD4^+^ and CD8^+^ T cell recognition (neo) antigens. In this method, known as *HANSolo* (HLA-Agnostic Neoantigen Screening), patient-matched Bcl-6/xL-immortalized B cell lines are constructed to express large libraries of minigenes that encode for screening T cell antigens. This approach provides enhanced sensitivity, particularly in the discovery of neoantigens recognized by CD4^+^ T cells, while enabling a significant increase in throughput [82]. With the development of single-cell RNA sequencing(scRNA-seq) and TCR sequencing, it has made it easier to obtain TCRαβ pairs from blood or tumor tissues. Accordingly, more platforms with distinct biological mechanisms are exploited to discover TCR ligands. Li et al. developed a cell-based selection platform for T cell antigen discovery by exploiting a membrane transfer phenomenon called trogocytosis. When T cells transfer N-hydroxysuccinimide (NHS)-biotin-labeled surface proteins to cognate target cells, the latter can be identified and sorted by flow cytometry for peptide sequencing [83]. Also, a chimeric receptor group, termed signaling and antigen-presenting bifunctional receptors (SABRs) provides a second cell-based platform for TCR ligand discovery. Extracellular domain of a SABR can be covalently linked to a peptide-β2 microglobulin-MHC trimer, which is further fused with an intracellular CD3ζ signaling domain. After interaction with a TCR, a SABR presenting its cognate antigen will induce GFP expression in NFAT-GFP-Jurkat cells upon receiving a signal of CD3ζ [84]. In addition, T-Scan, a high-throughput screening approach of TCR-recognized antigens, has been developed using a lentiviral delivery of antigen libraries with endogenous processing and presentation on MHC molecules. Target cells functionally recognized by T cells are isolated using a reporter for granzyme B activity and then antigens mediating recognition are identified by next-generation sequencing [85] (Fig. 4).

### Experimental strategies for neoantigen-reactive TCRs screening

In the screening of neoantigen-specific TCRs, peptide MHC tetramers (pMHC tetramers) and 4-1BB staining are widely used in multiple cancers, such as myeloma and metastatic urothelial carcinoma, etc. [86, 87]. Tetramers can simultaneously recognize a variety of antigen-specific T cells, but the types of MHC’s are limited (such as HLA-A*0101, A*0201, B*0702, B*0801, B*3501) due to their complicated synthesis technology [88]. Overall et al. used molecular chaperone TAPBPR for a stable capture of tetramerized empty MHC-I molecules, which can be readily loaded with interested peptides in a high-throughput manner [89]. At the same time, labeling tetramers with DNA barcodes and multiple fluorochromes significantly increase antigen species in one screening [89, 90]. Although widely used to detect response T cell populations, IFN-γELISPOT and cytotoxic activity lack sensitivity at single-cell level against neoantigens [77]. Some special biosensors, such as fluorescence resonance energy transfer (FRET), fluorescent NFAT and H2B histone sensors, have been designed to detect TCR activation [91–93]. However, the specificity of these signals in TCR screening is still questionable. A chemoenzymatic based platform called FucoID has been developed to anchor fucosyltransferase on the surface of dendritic cells (DCs). When DCs interacts with cognate TCR, biotin in the substrate of GDP-fucosylated-biotin can be transferred to T cell surface, enabling to distinct TSA-reactive T cells from bystander T cells in TILs [94]. Furthermore, a microfluidic-based screening system is used to encapsulate single TCR-T cell and single target cell with NFAT/AP-1-regulated eGFP in a well. If TCR-T cell interacts with its cognate antigen, fluorescence changes can be observed by microscopy [95, 96] (Fig. 4).

As the traditional TCR-T development process is time-consuming and inefficient, it may not be suitable for personalized therapy. With the help of scRNA-seq, TCR-seq, cellular indexing of transcriptomes and epitopes by sequencing (CITE-seq) and other technologies, researchers are able to comprehensively characterize T cells rapidly in a variety of tumors using a panel of CXCL13, ENTPD1(CD39) and CD200, the high-frequency molecular features of tumor neoantigen specific T (Tas) cells. Obtaining neoantigen-specific TCRs directly from patients can greatly accelerate the personalized T cell therapy [97–99]. For example, He et al. have established a complete technical platform for rapid TCR cloning and a personalized TCR-T therapy in phase I (NCT03891706) [97].

All these strategies are based on different principles and provide creative tools for screening of tumor neoantigen-responsive T cells.

## Neoantigen-targeted TCR-T therapy in clinical trials

Mutation neoantigens have greater individual differences and less potential epitopes than TAA or oncoviral antigens. Even the different types and quantities of neoantigens in different individuals of the same tumor caused by specificity of mutations showing obvious individual heterogenity. More and more neoantigen-based TCR-T clinical trials appear using "hot spot" mutants of oncogene or tumor suppressor gene [100]. For example, high affinities of TCRs against KRAS^G12D^ and KRAS^G12V^ variants have successfully been studied [101, 102]. After stimulation with mutated KRAS^G12D^ and KRAS^G12V^ in vitro, CD4^+^ and CD8^+^ memory T cells were identified in 3 of 6 metastatic cancer [103]. Leidner et al. reported the benefit of a single infusion of KRAS^G12D^-based TCR-T therapy in a patient with refractory recurrent pancreatic cancer. Although the patient failed to respond to surgery, neoadjuvant chemotherapy and autologous TILs therapy, produced regression of visceral metastases after treatment with HLA-C*08:02-restricted TCR-T cells for more than 6 months [104]. Similarly, *P53* mutants, such as R175H, Y220C and R248W are also immunogenic and can be recognized by T cells [105]. Kim et al. reported 97 of 163 patients with metastatic solid tumors had non-synonymous mutations of *P53* gene, 39 TCRs against 21 distinct *P*53 mutants were raised in TILs, and eventually, 2 of 12 individuals were in partial responses for 4 and 6 months respectively after treatment with *P53* mutant reactive TILs. When treated with autologous peripheral blood lymphocytes pre-transduced with an allogeneic HLA-A*02–restricted TCR specific for p53^R175H^, the patient with chemorefractory breast cancer experienced an improved immunophenotype, objective tumor regression (~ 55%) and prolonged survival over 6 months [106]. Diffuse intrinsic midline glioma (DIPG) is an aggressive childhood tumor of brainstem with no curative treatment available currently. Majority of DIPG’s often harbor an amino acid substitution from lysine(K) to methionine(M) at position 27 of histone 3 variant 3 (H3.3K27M mutation) which disrupts bivalent chromatin domains and drives neural stem cell-specific gliomagenesis [107, 108]. A TCR-T with HLA-A*02:01-restriction has been successfully constructed to recognize H3.3K27M. Adoptive transfer of these TCR-T cells significantly suppressed the progression of glioma xenografts in mice [107]. An early phase clinical study using TCR-T cells against H3.3K27M (NCT05478837) has been initiated in glioma patients (Table 3). At the same time, some clinical trials targeting personalized neoantigen TCR-T for different individuals in different solid tumors are also being recruited and conducted, such as NCT05194735, NCT03412877 (Table 3). Several personalized therapies against multiple targets are also being developed. In chemorefractory HR-positive metastatic breast cancer, mutated proteins identified by RNA sequencing and adoptive TILs transfer against mutant SLC3A2, KIAA0368, CADPS2 and CTSB with IL-2 and checkpoint blockade achieved complete tumor regression over 22 months [109]. Currently a clinic trial of engineered TCR-T cells targeting one to five neoantigens (NCT05349890) is going on with PD-1 inhibitors and CD4 agonists (Table 3). Although most TCR-T studies are still in the preclinical stage, it shows great potential to cancer patients with tumor-specific mutations.

**Table 3:** Clinical trials of engineered TCR-T cells for neoantigens (TCR-T trials registered in clinicalTrials.gov were summarized as of July 17th 2023.)

NCT Number	Title	Target neoantigen	Cancers	Interventions	Phases/Status
NCT05478837	Genetically Modified Cells (KIND T Cells) for the Treatment of HLA-A*0201-Positive Patients With H3.3K27M-Mutated Glioma	H3.3K27M	Diffuse Midline Glioma, H3 K27M-Mutant	Biological: Autologous Anti-H3.3K27M TCR-expressing T-cells	Phase 1/Not yet recruiting
				Drug: Cyclophosphamide,Fludarabine	
NCT05438667	TCR-T Cell Therapy on Advanced Pancreatic Cancer and Other Solid Tumors	KRAS G12V and G12D	Pancreatic Cancer	Biological: TCR-T therapy	Early Phase 1Recruiting
NCT05349890	Personalized TCR-T: Study of Adoptively Transferred T-cell Receptor Gene-engineered T Cells (TCR-T)	one to five tumor-specific neoantigens expressed by their autologous tumor	Malignant Epithelial Neoplasms	Biological: TCR-transduced T cells	Phase 1/Enrolling by invitation
				Drug: CDX-1140, Pembrolizumab	
NCT05194735	Phase I/II Study of Autologous T Cells to Express T-Cell Receptors (TCRs) in Subjects With Solid Tumors	unspecified	Gynecologic CancerColorectal Cancer|Pancreatic Cancer(and 9 more…)	Biological: Neoantigen specific TCR-T cell drug product, Aldesleukin (IL-2)	Phase 1|Phase 2/Recruiting
NCT04520711	Hotspot TCR-T: A Phase I/Ib Study of Adoptively Transferred T-cell Receptor Gene-engineered T Cells (TCR-T)	one to five tumor-specific neoantigens expressed by their autologous tumor	Malignant Epithelial Neoplasms	Biological: TCR-transduced T cells	Phase 1/Recruiting
				Drug: CDX-1140, Pembrolizumab	
NCT04146298	Mutant KRAS G12V-specific TCR Transduced T Cell Therapy for Advanced Pancreatic Cancer	KRAS G12V	Pancreatic Cancer|Pancreatic Neoplasms|Pancreatic Ductal Adenocarcinoma|Advanced Cancer	Biological: Mutant KRAS G12V-specific TCR transduced autologous T cells	Phase 1|Phase 2/Recruiting
				Drug: Cyclophosphamide, Fludarabine, Anti-PD-1 monoclonal antibody	
NCT03970382	A Study of Gene Edited Autologous Neoantigen Targeted TCR-T Cells With or Without Anti-PD-1 in Patients With Solid Tumors	unspecified	Solid Tumor	Biological: NeoTCR-P1 adoptive cell therapy, nivolumab, IL-2	Phase 1/Suspended
NCT03745326	Administering Peripheral Blood Lymphocytes Transduced With a Murine T-Cell Receptor Recognizing the G12D Variant of Mutated RAS in HLA-A*11:01 Patients	KRAS G12D	Gastrointestinal Cancer|Pancreatic Cancer|Gastric Cancer(and 2 more…)	Biological: anti-KRAS G12D mTCR PBL	Phase 1|Phase 2
				Drug: Cyclophosphamide, Fludarabine, Aldesleukin	
NCT03412877	Administration of Autologous T-Cells Genetically Engineered to Express T-Cell Receptors Reactive Against Neoantigens in People With Metastatic Cancer	unspecified	Endocrine Tumors|Non-Small Cell Lung Cancer|Ovarian Cancer(and 4 more…)	Biological: Individual Patient TCR-Transduced PBL	Phase 2/Recruiting
				Drug: Cyclophosphamide, Fludarabine, Aldesleukin, Pembrolizumab (KEYTRUDA)	
NCT03190941	Administering Peripheral Blood Lymphocytes Transduced With a Murine T-Cell Receptor Recognizing the G12V Variant of Mutated RAS in HLA-A*11:01 Patients	KRAS G12V	Pancreatic Cancer |Gastric Cancer |Gastrointestinal Cancer|Colon Cancer|Rectal Cancer	Biological: Anti-KRAS G12V mTCR PBL	Phase 1|Phase 2/Recruiting
				Drug: Cyclophosphamide,Fludarabine,High-dose Aldesleukin	
NCT00704938	Gene-Modified Lymphocytes, High-Dose Aldesleukin, and Vaccine Therapy in Treating Patients With Progressive or Recurrent Metastatic Cancer	P53	Kidney Cancer|Melanoma (Skin)|Unspecified Adult Solid Tumor,Protocol Specific	Biological: aldesleukin, anti-p53 T-cell receptor-transduced peripheral blood lymphocytes, autologous dendritic cell-adenovirus p53 vaccine,filgrastim	Phase 2
				Drug: cyclophosphamide,fludarabine phosphate	
NCT00393029	Phase II Study of Metastatic Cancer That Overexpresses P53 Using Lymphodepleting Conditioning Followed by Infusion of Anti-P53 TCR-Gene Engineered Lymphocytes	P53	Metastatic cancer	Biological: anti-protein 53 or tumor protein 53 (p53) T-cell receptor transduced peripheral blood lymphocytes, aldesleukin,filgrastim	Phase 2/Completed
				Drug: cyclophosphamide,fludarabine phosphate	
NCT03431311	T Cell Receptor Based Therapy of Metastatic Colorectal Cancer	TGFβII (frameshitf)	Colorectal Cancer	Biological: Adoptive Cell Therapy	Phase 1|Phase 2/Terminated
NCT04102436	Non-Viral TCR Gene Therapy	Unspecified	Glioblastoma|Non-Small Cell Lung Cancer|Breast Cancer|Gastrointestinal/Genitourinary Cancer|Ovarian Cancer	Biological: Sleeping Beauty Transposed PBL	Phase 2/Suspended
				Drug: Fludarabine,Cyclophosphamide,Aldesleukin	
NCT03891706	Individualized Tumor Specific TCR- T Cells in the Treatment of Advanced Solid Tumors	unspecified	Solid Tumor	Drug: tumor-specific TCR-T cells; Interleukin-2	Phase1/Recruiting

*Abbreviations* H3.3K27M, a Lys 27-to-methionine (K27M) mutation at histone H3 variant H3.3; KRAS, kirsten rat sarcoma viral oncogene homolog; IL-2, interleukin-2; NeoTCR P1, is composed of apheresis derived CD8 and CD4 T cells that are precision genome engineered to express one autologous TCR of native sequence that targets a neoepitope (neoE) presented by human leukocyte antigen (HLA) receptors exclusively on the surface of that patient's tumor cells and not on other cells in the body; PBL, peripheral blood lymphocytes; p53,tumor protein 53; TGFβII, transforming growth factor-β isoform 2

In addition, single nucleotide variants (SNVs), antigens derived from frameshifts, splice variants, gene fusions, and endogenous retroelements have been recently evaluated (Table 1). And several personalized therapies against non-coding genes-derived peptides are also being developed as an alternative source of neoantigens [110].

## Challenges of neoantigen-based TCR-T therapies

Although less cytokine release syndrome and neurotoxicity were expected in TCR-T cell therapy than CAR-T cell therapy due to its specific expression of neoantigens in tumor tissue, many challenges and limitations remain in its practical application, such as tumor cell heterogeneity, the mismatched pair of exogenous TCRs with endogenous TCRs, the durability of engineered TCR-T cells in vivo and the untoward effects of immunosuppressive tumor microenvironment.

### Tumor heterogeneity

Intratumor heterogeneity (ITH), that is, clonal diversity of subclonal cell populations within a tumor. The higher ITH tumors have weaker antitumor immune responses and more susceptibility to progression. In more heterogeneous tumor cell populations, tumor cells could have a better chance of escaping immune surveillance because the reactive neoantigens undergo “dilution” within the tumor relative to other neoantigens [111, 112]. On the one hand, this results in a more complex neoantigen prediction progress, and the frequency of neoantigen-specific T cells in TILs is lower. On the other hand, antigen loss and down-regulation of MHC-I/II molecules in some subclonal populations often occur with the tumor progression or after targeted immunotherapy. At the same time, cytotoxic T cell loss the capacity to kill tumor cells that are deficiencies in antigen-presentation process [113, 114]. The implication, however, is that TCR-T therapy for a single target may not be effective in tumor killing.

There have some strategies to target ITH in TCR-T therapies. Oncolytic viruses, chemotherapeutic drugs and radiation therapy could induce immunogenic cell death (ICD) [115]. Lysis of tumor cells can release abundant amounts of antigens and cytokines within the TME, resulting in the activation of potent, multiepitope immune response [116]. In the selection of TCR-T targets, the neoantigens encoded by hotspot mutations in driver genes may be prioritized. Such antigens are usually necessary for tumor progression and are less prone to natural loss (discussed in more detail in Sect. Neoantigen-targeted TCR-T therapy in clinical trials). Moreover, the natural TCR structure were modified to overcome the down-regulation of MHC molecules (discussed in more detail in Sect. Recombinant TCRs). And unlike classical αβT cells, γδT cells are not restricted to pMHCs, and the natural killer cell receptors (NKRs) expressed can identify stress antigens that are upregulated in many tumor types [117]. Recently, a study by de Vries et al. revealed that γδT cells are effector cells of immunotherapy in DNA mismatch repair-deficient (MMR-d) cancers, and *B2M* inactivating mutations can activate γδT cells. What’s more, these γδT cells are mainly composed of Vδ 1 and Vδ 3 isoforms with strong killing activity [118]. These researches indicated that γδT cells may be suitable for the treatment of MHC-deficient tumors, as well as for their application in allogeneic cell therapy.

### Limitations of neoantigen prediction accuracy

Over the past few decades, the process of identifying neoantigens has continually evolved and improved, and it has also found widespread application in clinical trials. But developing a precise and robust pipeline for identifying neoantigens is often complex, requiring high-quality DNA/RNA sequencing data and corresponding mass spectrometry data, as well as rigorous testing and training of high-precision algorithms. Previous studies indicate that only about 1% of mutations give rise to neoantigens that elicit spontaneous TIL responses [119]. Among the reasons why T cells may not recognize enough neoantigens are the similarities between mutant-peptides and wild- peptides, as well as between the functional T-cell receptor repertoire induced by central or peripheral tolerance mechanisms [120]. Furthermore, the currently developed neoantigen prediction pipelines lack specific prediction tools to support neoantigen prediction beyond SNVs and Indels, such as RNA splicing and transcriptome alternative splicing. At present, the accuracy of neoantigen prediction is less than 50% [121]. Therefore, how to quickly verify whether the predicted tumor epitopes are clinically applicable neoantigens will still remain a bottleneck in the near future.

### Delivery system

Currently most engineered TCR are delivered by lentivirus whose biosafety is not fully understood in patients. Insertion mutations, shedding and immunogenicity may occur due to its random integration in chromosomes, resulting in defective gene expression and even oncogene activation in T cells [122]. Therefore, it is critical to find more effective and safer vectors instead of lentivirus. Through a molecular cut-and-paste mechanism, the Sleeping Beauty (SB) transposition system has be used in specific-site genetic operation by recognizing inverted terminal repeats (ITRs) [123]. A clinical trial of SB-modified CAR-T cells against B-cell malignant lymphomas has been reported [123]. Constructed TCR-T cells using SB transposon system did elicit immune reaction against mutated neoantigen in tumor cell lines [124]. Specific TCR modified by SB has a good prospect for personalized T cell immunotherapy, considering its low cost, fast in production, non-viral vector and high biosafety (Table 3). Other technologies as CRISPR/cas9 and nucleases are also trying as non-viral vectors in TCR-T construction [125, 126].

However, the complexity of customizing engineered T-cells ex vivo and the resulting reduction in T-cell viability and efficacy can be prohibitive for extending to different types of tumors and diverse patient populations. Researchers have found that combining nanotechnology approaches can help mitigate these limitations in TCR or costimulatory signals delivery. Multifunctional nanoparticles can directly modulate receptor clusters to enhance the delivery efficiency of TCR while reducing off-target toxicity [127]. Parayath et al. demonstrated the use of biodegradable polymer nanocarriers to deliver in vitro-transcribed (IVT) CAR or TCR mRNA for transiently reprogramming circulating T-cells to recognize disease-related antigens [128]. Perica et al. showed that stimulating anti-tumor activity can be achieved by presenting pMHC to relevant TCRs, with magnetic nanoparticle carriers enhancing the strength of antigen-specific T-cells [129]. In addition, nanotechnology can achieve high drug loading and controlled drug delivery at tumor sites. For example, Tang et al. developed protein-based nanogel particles (NGs) that can precisely control cytokine release and selectively activate immune cells in tumor microenvironment. These NGs function as "nanoscale backpacks" comprised of many copies of the protein crosslinked to itself (self-assembled nanoparticles), thereby achieving carrier-free cytokine delivery that increase the efficacy and safety [130]. Moreover, several recent developed nanomaterial-based strategies could control the nanoscale distribution of immunoregulatory agents and regulate T cell behavior, such as biomimetic modified nanoparticles [131], deformable nanoparticles [132], photothermal effect nanoparticles [133], stimuli-responsive nanoparticles [134], etc. Linking drug delivery to TCR activation through nanotechnology holds great promise for T cell-based immunotherapy in the field of cancer immunotherapy.

### TCR mismatch

As mentioned above, most T cells express αβTCR and few express γδTCR [6]. When T cells engineered with exogenous αβTCR, mismatched TCRs between exogenous and endogenous alpha/ beta chains may occur, and vice versa [135]. In mice studies, infusing TCR-T cells with mismatched novel TCRs may lead to severe graft versus host disease (GVHD) [136]. In addition, exogenous TCRs, endogenous TCRs and mismatched TCRs will compete with CD3 molecules for T cell signaling, resulting in a decrease expression of exogenous TCR, inefficient T cell activation and reduced T cell cytotoxicity [137]. Therefore, mismatch of TCRs must be prevented.

CRISPR/CAS9 has been used to edit TRAC and TRBC gene loci, to knockout endogenous α and β encoding genes and to increase the expression of exogenous TCR. A pre-clinical study revealed an enhanced T cell recognition of multiple myeloma and prolong survival of tumor-burdened mice using these modified T cells [138]. More recently, another clinical-grade approach with CRISPR/Cas9 system to knockout the endogenous TRAC and TRBC genes and insert transgenic neoantigen-specific TCR (neoTCR) into the TRAC locus was described by Foy et al. The dose escalation of neoTCR-T cells was initiated presently in phase I clinical trial (NCT0370382) [139].

Introducing αβTCR into γδT cells can significantly reduce TCR pairing errors. No mismatch between γδTCR and αβTCR and no cytotoxic activity of normal cells were found in γ4δ1 T cells transferred with TCRαβ [140]. Moreover, the constant region of human TCR can be replaced by that of the mouse. Additionally, a cysteine mutation is introduced to stabilize the entire TCR receptor through disulfide bonds which can reduce the binding affinity to the endogenous TCRa/β chain [10].

### Immunosuppressive tumor microenvironment

Tumor microenvironments (TME) have been confirmed to play a pivotal role not only in tumorigenesis and progression but also on T cell proliferation and function. The dysfunction of T cells in TME usually lead to the failure of TCR-T therapy. Therefore, remodeling the immune microenvironment is an important strategy to improve the efficacy of immunotherapy. The following factors should be especially considered during TCR-T therapy:

1. Chemokines

Increasing chemotaxis and its signaling of immunoreactive cells is a major strategy in TME remodeling. Highly expressed CXCL9/10 /11 may help effector T cell migration and infiltration into tumor tissues [141]. Up-regulation of CXCR2 can improve migration of TCR-T cells to tumor tissues [142, 143]. Chemokine-antibody fusion proteins enhances intratumoral recruitment of effector T cells by directly targeting the chemokine of tumor cells. For example, a glioma targeting fusion protein of CXCL10-EGFRvIII scFv was constructed and tested in combination with tumor antigen-specific CD8^+^ T cells [144]. More recently, Tian et al. generated OV-Cmab-CCL5 by oncolytic herpes simplex virus type 1 (oHSV), in which a secretable single-chain variable fragment of the EGFR antibody (cetuximab) was linked to CCL5 using Fc knob-into-hole strategy. Due to the continuous production of CCL5 in TME, OV-Cmab-CCL5 significantly enhances the migration and the activation of natural killer cells, macrophages and T cells [145]. The synergistic effects of chemokines may enhance the therapeutic efficiency of TCR-T cells in clinic.

2) Metabolites

Metabolites in TME can moderate the anti-tumor immune response. Notarangelo et al. recently reported that D-2-hydroxyglutarate (D-2HG), a metabolite of tumors with mutated isocitrate dehydrogenase (IDH), impairs CD8^+^ T cell mediated tumor cell cytotoxicity. Overaccumulation of D-2HG in TME would inhibit lactate dehydrogenase (LDH) activity and glucose metabolism, thus damage the activation, proliferation and cytotoxicity CD8^+^ T cells [146]. Cheng et al. reported that mutation or depletion of fumarate hydratase (FH) in tumor cells accumulated fumarate in tumor interstitial fluid, impairing TCR signaling by succinating ZAP70 at C96 and C102, and subsequently, dampening the anti-tumor responses of infiltrating CD8^+^ T cells. Removal of fumarate by FH reexpression significantly enhanced anti-CD19 CAR-T efficiency in xenograft tumor model [147]. Therefore, modulation of oncometabolites in TME may be an important strategy to improve tumor immunotherapy. As metabolites are numerous and dynamic, it is difficult to specify which one is most suitable in TME remodeling.

3) Checkpoint molecules

Immune checkpoints refer to the receptors and corresponding ligands that can positively or negatively regulate T cell activation. For example, CD40 is expressed in a variety of immune system cells including antigen-presenting cells and its ligand, CD40L, is transiently expressed on the surface of activated T cells. CD40/CD40L signaling "permits" dendritic cells to mature and then trigger T cell activation and differentiation. Inhibitory receptors such as PD-1, CTLA-4 in activated T cells interact with PD-L1, CD80/86 respectively in tumor cells or stromal cells, transmit immunosuppressive signals, induce T cell apoptosis and inhibit T cell function [148]. Most recently, a clinical trial has begun to determine the safety and the objective response of adoptive TCR-T transfer against TSA in combination with CD40 (CDX-1140) and PD-1(pembrolizumab)(NCT05349890). A number of bispecific antibodies which block PD-L1/LAG-3 and other targets (e.g., PD-1/VEGF) have emerged [149, 150]. Of course, more clinical trials are needed to explore the efficacy and side effects of these modulators in different panels of combinations.

## Future directions

It is well known that T cells are composed of multiple subpopulations, including CD8^+^ T cells, CD4^+^ T cells and Tregs. By lineage analysis, T cells can also be divided into naive T cells (T_N_), stem cell memory T cells (T_SCM_), central memory T cells(T_CM_) and effector T cells(T_EFF_) [151]. The synergistic effects of a combined use of appropriate T subsets might improve efficacy of TCR-T immunotherapy. In a clinical trial, 32 patients of non-Hodgkin B-cell lymphoma treated with CD19 CAR-T cells in a 1:1 ratio of CD8^+^/CD4^+^ T cells exhibit long duration of CAR-T cells and have slow disease progression [152]. Cachot et al. showed the dual functions of cytotoxicity and immunoregulation of tumor-specific CD4^+^ T cells, especially on MHC-I loss or down-regulation tumor cells [153]. Theoretically, recruiting an appropriate proportion of CD4^+^ cells may improve therapeutic effects of TCR-T cells. In addition, memory T cells are confirmed to have superior anti-tumor effects because of their long duration, strong homing ability to lymph nodes and lower threshold for antigen activation than naive T cells [151]. Adding IL-15 and IL-21 can elevate gp100 targeting TCR-T effects by 10-100 times due to a successful induction of T_SCM_ or T_CM_ from T_N_ cells [154–156]. Glycogen synthase-3β inhibitor TWS119 can better induce T_N_ and obtain T_SCM_ of clinically available magnitude [157]. And mitochondrial pyruvate carrier (MPC) inhibitor favors memory T cell differentiation with a superior and long-lasting anti-tumor activity in tumor model [158]. Currently, the generation of a massive number of T cells that provide long-lasting immunity is challenged not only by the quality of patient tissue source, but also the exhaustion and differentiation-associated senescence which arise during in vitro cloning and expansion. To address these problems, several studies have developed a strategy to regenerated cytotoxic T lymphocytes (CTL) from induced pluripotent stem cells (iPSCs) through transduction of TCR to clinical-grade HLA-haplotype homozygous iPSCs [159, 160]. Kawai et al. demonstrated that the modified iPSC-CTLs exhibited early memory phenotype, including high replicative capacity and the ability to give rise to potent effector cells [161].

Recently, myeloid cells have been found to promote T cell functions in tumor immunotherapy. Anti-tumor neutrophil subsets were observed both in mouse and human biopsies after immune therapies [162, 163]. Hirschhorn et al. showed that melanoma-specific CD4^+^ T cells in combination with OX40 co-stimulation or CTLA-4 blockade can eradicate melanomas containing antigen escape variants. In this scenario, CD4^+^ T cells play on-target cytotoxicity of melanoma while neutrophils are responsible for killing antigen loss variants [163]. Linde et al. also demonstrated that neutrophils can be activated and kill tumor cells in combined of tumor necrosis factor, CD40 agonist and tumor-binding antibody in vitro and in vivo model [164]. All these data suggest the auxiliary role of neutrophils in improving adoptive T cell therapy. Besides, as discussed above, the heterogeneity of solid tumors, loss of neoantigen expression or dysfunction of tumor antigen presentation usually lead to the failure of T cell immunotherapy [165, 166], which can be partially reversed by chemoradiotherapy [167, 168]. Furthermore, adoptive T cell therapy combined with anti-angiogenic drugs [169], oncolytic viruses [170], neoantigen vaccine [171],etc., should be considered and tested in the future.

## Conclusion

Discovery of neoantigens and their specific TCR repertoires is the key step for a successful TCR-T based immunotherapy. With the great advances of omics research technologies, more and more neoantigens are available for personalized immunotherapy through experimental and bioinformatic analysis. A combined strategy of computational prediction and experimental validation in neoantigens and their cognate TCR screening is encouraged for its time-saving, efficient and practiced in clinic. Tumor heterogeneity and tumor immunosuppressive microenvironment are still the two main challenges in neoantigen based TCR-T therapies in use for a relatively long period. And finally, TCR-T cells with other therapies should be a right direction in the future.

## Data Availability

Not applicable.

## Abbreviations

TCR-T: T cell receptor-engineered T cell therapy
ACTs: Adoptive cell therapies
TAAs: Tumor-associated antigens
TSAs: Tumor-specific antigens
CD3: Cluster of differentiation 3
MHC: Major histocompatibility complex
ITAM: Immunoreceptor tyrosine-based activation motif
CS: Cholesterol sulfate
pMHCs: Peptide major histocompatibility complexes
APCs: Antigen-presenting cells
IK: Immunological kinapse
IS: Immunological synapse
CAR: Chimeric antigen receptor
STAR-T: Synthetic T cell receptor and antigen receptor-T
hSTAR: Human TCR constant region-based STAR
TRuCs: T cell receptor fusion constructs
scFv: Single-chain variable fragment
TCRm: TCR mimic
AbTCR: Antibody-T cell receptor
Glypican-3: GPC-3
ImmTACs: Immune-mobilizing monoclonal T cell receptors against cancer
ROC: Receiver operating characteristic
MS: Mass spectrometry
CDR: TCR complementarity determining region
NLP: Natural Language Processing
AE: Autoencoder
LSTM: Long short-term memory
imRex: Interaction map recognition
TAP: Transporter associated with antigen processing
WES: Whole-exome sequencing
SLP: Synthetic long peptide
ELISPOT: Enzyme-linked immunospot
ccRCC: Clear cell renal cell carcinoma
TMG: Tandem minigene
TILs: Tumor infiltrating lymphocytes
HANSolo: HLA-Agnostic Neoantigen Screening
scRNA-seq: Single-cell RNA sequencing
SABRs: Signaling and Antigen-presenting Bifunctional Receptors
pMHC tetramers: Peptide MHC tetramers
FRET: Fluorescence resonance energy transfer
DCs: Dendritic cells
CITE-seq: Cellular indexing of transcriptomes and epitopes by sequencing
Tas: Tumor neoantigen specific T
DIPG: Diffuse intrinsic midline gliomas
SNVs: Single nucleotide variants
ITH: Intratumor heterogeneity
ICD: Immunogenic cell death
NKRs: Natural killer cell receptors
MMR-d: DNA mismatch repair-deficient
SB: Sleeping Beauty
ITRs: Inverted terminal repeats
IVT: In vitro-transcribed
NGs: Protein-based nanogel particles
GVHD: Graft versus host disease
neoTCR: Neoantigen-specific TCR
TME: Tumor microenvironment
oHSV: Oncolytic herpes simplex virus type1
IDH: Isocitrate dehydrogenase
LDH: Lactate dehydrogenase
FH: Fumarate hydratase
T_N_: Naive T cells
T_SCM_: Stem cell memory T cells
T_CM_: Central memory T cells
T_EFF_: Effector T cells
MPC: Mitochondrial pyruvate carrier
CTL: Cytotoxic T lymphocytes
iPSCs: Induced pluripotent stem cells
